# The impact of inspired oxygen levels on calibrated fMRI measurements of *M*, OEF and resting CMRO_2_ using combined hypercapnia and hyperoxia

**DOI:** 10.1371/journal.pone.0174932

**Published:** 2017-03-31

**Authors:** Isabelle Lajoie, Felipe B. Tancredi, Richard D. Hoge

**Affiliations:** 1 Département de physiologie moléculaire et intégrative, Institut de génie biomédical, Université de Montréal, Montreal, Quebec, Canada; 2 Department of Neurology and Neurosurgery, Montreal Neurological Institute, McGill University, Montreal, Quebec, Canada; 3 Departamento de Radiologia, Centro de Pesquisa em Imagem, Hospital Israelita Albert Einstein, São Palo, SP, Brazil; Penn State University, UNITED STATES

## Abstract

Recent calibrated fMRI techniques using combined hypercapnia and hyperoxia allow the mapping of resting cerebral metabolic rate of oxygen (CMRO_2_) in absolute units, oxygen extraction fraction (OEF) and calibration parameter *M* (maximum BOLD). The adoption of such technique necessitates knowledge about the precision and accuracy of the model-derived parameters. One of the factors that may impact the precision and accuracy is the level of oxygen provided during periods of hyperoxia (HO). A high level of oxygen may bring the BOLD responses closer to the maximum *M* value, and hence reduce the error associated with the *M* interpolation. However, an increased concentration of paramagnetic oxygen in the inhaled air may result in a larger susceptibility area around the frontal sinuses and nasal cavity. Additionally, a higher O_2_ level may generate a larger arterial blood *T*_1_ shortening, which require a bigger cerebral blood flow (CBF) *T*_1_ correction. To evaluate the impact of inspired oxygen levels on *M*, OEF and CMRO_2_ estimates, a cohort of six healthy adults underwent two different protocols: one where 60% of O_2_ was administered during HO (low HO or LHO) and one where 100% O_2_ was administered (high HO or HHO). The QUantitative O2 (QUO2) MRI approach was employed, where CBF and R2* are simultaneously acquired during periods of hypercapnia (HC) and hyperoxia, using a clinical 3 T scanner. Scan sessions were repeated to assess repeatability of results at the different O_2_ levels. Our *T*_1_ values during periods of hyperoxia were estimated based on an empirical ex-vivo relationship between *T*_1_ and the arterial partial pressure of O_2_. As expected, our *T*_1_ estimates revealed a larger *T*_1_ shortening in arterial blood when administering 100% O_2_ relative to 60% O_2_ (*T*_1LHO_ = 1.56±0.01 sec vs. *T*_1HHO_ = 1.47±0.01 sec, *P* < 4*10^−13^). In regard to the susceptibility artifacts, the patterns and number of affected voxels were comparable irrespective of the O_2_ concentration. Finally, the model-derived estimates were consistent regardless of the HO levels, indicating that the different effects are adequately accounted for within the model.

## Introduction

Recently, different groups have proposed that resting cerebral metabolic rate of O_2_ consumption (CMRO_2_) can be imaged using gas-based fMRI techniques [1–3]. Our team presented an approach, dubbed QUantitative O_2_ (QUO2) based on respiratory calibration of the BOLD signal, using hypercapnia (HC), and hyperoxia (HO). During the gas manipulation, end-tidal O_2_ (ETO_2_) and CO_2_ (ETCO_2_) levels are constantly monitored and a dual-echo version of pseudo-continuous Arterial Spin Labeling (de-pCASL) is used to measure BOLD and cerebral blood flow (CBF) simultaneously. ETO_2_, BOLD and CBF then serve as inputs to the generalized calibration model (GCM) described in Gauthier and Hoge [4], which yields a system of two equations with solutions for the BOLD calibration parameter *M*, i.e. the maximum BOLD signal increase when venous O_2_ saturation approaches 100%, and resting oxygen extraction fraction (OEF). The multiplication of OEF by baseline CBF and arterial O_2_ content (estimated from ETO_2_ monitoring and, optionally, blood testing) gives the estimated resting CMRO_2_ in micromoles of oxygen extracted from the cerebral vasculature per minute, per 100g of tissue.

While the initial proof-of-concept of the method produced reliable results when spatially averaged within the brain and over multiple subjects, it suffered from a single-subject instability characterized by large fluctuations in the modeled values and a considerable lack of solution in certain regions [1]. In order to be considered a reliable method for within-subject longitudinal studies, there was a need to improve the single-subject image quality. Additionally, prior to being able to draw conclusion about differences in resting oxidative metabolism between populations or between states of a disease, knowledge about the precision and accuracy of the model-derived estimates was crucial. The breathing circuit and image analysis strategy were updated in previous work [5–6]. The repeatability of the respiratory responses as well as CBF and BOLD responses within gray matter (GM) has also been assessed [7]. Finally, the question of methodological precision was evaluated by assessing the regional intra- and inter-subject variability of QUO2 derived estimates [6].

The choice of O_2_ and CO_2_ concentration during respective periods of HO and HC may also have an impact on the accuracy and precision of QUO2 derived estimates, which remains to be assessed. Higher CO_2_ concentration would have the advantage of increasing the image contrast-to-noise ratio due to higher CBF responses, however it can lead to anxiety and potentially alter brain physiology in ways other than the intended vasodilatory effect [8,9]. In a preliminary phase, it was agreed that the commonly employed 5% CO_2_ during HC blocks was low enough to preserve participant’s comfort, while high enough to yield significant cerebrovascular responses. As for the O_2_ concentration, compared to slight HO levels (e.g. 50–60%), more extreme levels of HO may bring the BOLD responses closer to the maximum *M* value, therefore diminishing the measurement errors while increasing the SNR. However, due to the paramagnetic characteristic of oxygen molecule, the measured signal may be prone to more prominent susceptibility artifacts patterns in vulnerable regions such as the frontal sinuses and nasal cavity, thus yielding inaccurate or non-solution values in those regions. An additional potential impact of the O_2_ concentration arises when changes in blood flow during HO are encompassed in the model, such as in the generalized calibrated model. Following a low HO level, CBF responses may be smaller than the inherent noise level of ASL acquisitions, making its measurement challenging. Furthermore, a decrease in CBF during periods of HO may reflect a combination of phenomena: a vasoconstrictive effect following a hyperventilation-induced decrease in ETCO_2_ [10], a vasoconstriction due to increased O_2_ per se, and an acceleration of arterial blood longitudinal relaxation (*T*_1_ shortening) caused by the increase of dissolved molecular oxygen in blood plasma [11–14]. If not taken into account, this *T*_1_ decay in arterial blood leads to an overestimation of CBF decrease during HO. As a consequence of those complications, it is common to assume a fixed, pre-determined CBF decrease [2,15–17]. However, assuming a fixed CBF decrease contributes to the systematic errors and can affect the accuracy and repeatability of OEF and CMRO_2_ estimates as reported in Lajoie et al [6]. Therefore, the application of a *T*_1_-correction on the measured CBF during HO is advocated.

Additionally, in theory, the QUO2-derived estimates should not depend on the level of hyperoxia induced, since the model is designed to account for this. In a previous study [6], the within-subject repeatability of the model-derived estimates was assessed based on very small variations of ETO_2_ during periods of 60% O_2_ hyperoxia. The effectiveness of the QUO2 model to obtain reproducible *M*, OEF and CMRO_2_ despite considerable variations in hyperoxia ETO_2_ is crucial and remains to be demonstrated.

The present study aims at exploring, in a small cohort of healthy individuals, the impact mentioned above, on QUO2 calibrated fMRI estimates, when providing 100% O_2_ during periods of HO instead of the previously provided 60% O_2_, in addition to verifying the reproducibility of results regardless of the inspired oxygen levels.

## Materials and methods

From the group of eight healthy adults that underwent the 24 hour QUO2 test-retest study mentioned previously [6], six of them repeated the experiment, but this time, instead of being given 60% O_2_ during periods of HO (referred to as “lower HO levels protocol” (LHO)), the participants were given 100% O_2_ (“higher HO levels protocol” (HHO)). Each HO protocol was repeated to assess repeatability of results at the different O_2_ levels (referred to as “Test A” and “Test B”). To minimize effects of diurnal fluctuation in blood flow [18], all sessions were acquired between 2 PM and 6 PM. The participants were asked to abstain from caffeine 3 hours prior to scanning. All participants (3 females and 3 males, mean age: 30.5 ± 6.7 years) gave written informed consent and the project was approved by the Comité mixte d’éthique de la recherche du Regroupement Neuroimagerie/Québec.

### Respiratory paradigm

A gas timing schedule previously described by Bulte et al [2], with a total duration of 18 minutes, was applied, as in [6]. This involves two 2-min periods of hypercapnia (HC) and two 3-min periods of hyperoxia (HO), induced by administering gas mixtures enriched with CO_2_ and O_2_ respectively. Hypercapnia was followed by a 1-min normocapnic period and then the 3-min hyperoxic stimulus. Hyperoxia was followed by a 3-min period of normoxia. Periods of normocapnia and normoxia were long enough to ensure a return to baseline as shown by the CBF and BOLD time course in Tancredi et al, figure 3 [7]. Participants inhaled the gas mixtures via a breathing circuit developed in-house [5]. During the first test-retest experiment [6], the hyperoxia periods were induced with the subjects breathing a mixture of 50% pure oxygen balanced with air, yielding a fix inspired O_2_ concentration of 60% O_2_. During the second test-retest experiment, the participants were given 100% O_2_ during periods of HO. Otherwise participants were given medical air to breath. Respiratory gases were continuously monitored using the CO2100C and O2100C modules of a BIOPAC MP150 system (BIOPAC Systems Inc., CA, USA). For additional details, see Lajoie et al. [6].

### Image acquisition

Images were acquired on a clinical 3T MRI scanner (Siemens TIM TRIO, Siemens Medical Solutions, Erlangen, Germany) using the vendor’s 32-channel receive-only head coil. The scan session included a 5-minute anatomical acquisition (1 mm^3^ MPRAGE with TR/TE/flip angle = 2.3 seconds/3 msec/9°, 256x240 matrix, GRAPPA factor = 2), and an 18-minute functional scan using dual-echo pseudo-continuous ASL sequence (de-pCASL) [19] in order to acquire simultaneous measures of BOLD and CBF. The de-pCASL parameters were: TR/TE1/TE2/α = 4.12 seconds/8.4 msec/30 msec/90°, labeling duration = 2 seconds using Hanning window-shaped RF pulse with duration/space = 500 μsec/360 μsec, flip angle = 25°, peak gradient amplitude = 6 mT/m, mean gradient amplitude = 0.6 mT/m, label offset = 100 mm below the center of image slab, nominal and average post-labeling delay (PLD) = 0.9 and 1.44 seconds. The readout consisted of a GRE-EPI with GRAPPA factor = 2, partial sampling of k-space = 7/8, in-plane resolution of 4.5 x 4.5 mm^2^, 21 slices with 4.5 mm thickness and 0.45 mm gap.

### Respiratory data analysis

Analysis of the respiratory data was carried out using an in-house program developed in Matlab (MathWorks, Natick, MA, USA), as in Lajoie et al [6]. An automatic extraction of the end-tidal (ET) and end-inspiratory points from the continuous O_2_ and CO_2_ traces was performed. Each ET point was corrected to account for the low-pass filtering effect of the filter placed in series and to account for an expired partial pressure of water of 47 mmHg [20]. More details about the respiratory data analysis can be found in Lajoie et al [6].

The average values of ETO_2_ at baseline and during both respiratory stimuli were used to compute arterial O_2_ content (ml O_2_/ml blood) and change in the venous deoxygenated fraction ([dHb]/[dHb]_0_) as in Chiarelli et al [14] and Gauthier et al [1]. The latter quantities are needed to obtain the BOLD calibrated value *M*, resting OEF and CMRO_2_ as specified below.

### Imaging data analysis

#### Preprocessing

Analysis of functional scans along with exclusion of artifact and non-paranchymal voxels were performed using in-house software implemented in C, as in Lajoie et al [6].

During hyperoxic manipulation, the longitudinal relaxation time (*T*_1_) of blood is altered due to an increase in plasma concentration of paramagnetic O_2_ [13]. To account for this change in blood *T*_1_, that would bias the measured CBF changes, a corrective factor using the approach described in Chalela et al [21] and Zaharchuk et al [22] was applied. First, estimates of the arterial blood *T*_1_ values during hyperoxic periods were obtained based on the individual ETO_2_ measurements, used as a surrogate for arterial partial pressure of O_2_ (PaO_2_), along with the R1 (1/ *T*_1_) and PaO_2_ relationship in rats’ blood reported in Pilkinton et al [13]. Depending on whether our ETO_2_ values were within or outside the range of values in Pilkinton et al’s study, the *T*_1_ values were either linearly interpolated or extrapolated. Then, the individual blood flow maps during HO were corrected by applying a slice-wise corrective factor based on the quantitative blood flow equation [23], the slice acquisition time and the adjusted *T*_1_ value.

#### Computation of CMRO_2_

MRI measures of BOLD and CBF acquired during the hypercapnic manipulation, along with the changes in the venous deoxygenated fraction were used as inputs to the generalized calibration model (GCM), described in Gauthier and Hoge [4], yielding a functional curve (the “HC curve”) of possible pairings of *M* and OEF. Repeating the procedure with the hyperoxia measurements yielded a second curve of possible *M* and OEF pairings (the “HO curve”). The intersection of these two curves provided the true values of *M* and OEF at each voxel. Finally, CMRO_2_ was determined by multiplying OEF by O_2_ delivery, computed as the product of resting CBF by arterial O_2_ content. Since the small regional CBF responses to hyperoxia are difficult to measure due to the low SNR of ASL, a uniform change of CBF was assumed throughout the brain, based on the cortical gray matter change after *T*_1_ correction. Additional information about the computation of CMRO_2_ can be found in Lajoie et al [6].

#### Tissue segmentation

Automated segmentation of GM from the anatomical scans was carried out using the FMRIB Software Library (FSL) [24]. Structural images were extracted from *T*_1_-weighted scans using the brain extraction tool (FSL’s BET). Finally, a probability mask of GM was created employing the automated segmentation tool (FSL’s FAST), and was resampled to the resolution of the functional EPI scans.

#### Regions Of Interest (ROIs)

**Fig 1:**
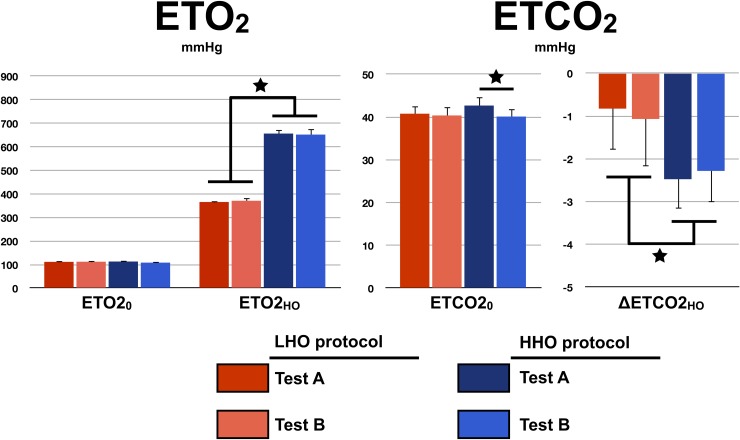
Gas manipulation. For each protocol and test, the measured resting (with the subscript ‘0’) and hyperoxic (with the subscript ‘HO’) end-tidal O_2_ and CO_2_ are presented. Errors bars indicate standard deviation. A star indicates a significant difference at *P* < 0.05.

The model-derived estimates were evaluated throughout cortical GM as well as within six ROIs selected from the ICBM OASIS-TRT-20 atlas [25] and presented in Lajoie et al [6], figure 1: the inferior parietal, superior parietal, precuneus, hippocampus, anterior (caudal and rostral) cingulate and posterior cingulate. Each ICBM three-dimensional ROI was registered to the resolution of the functional EPI scans before being conjoined with the individual’s GM probability mask excluding voxels with a GM probability lower than 50% as well as non-parenchymal voxels previously identified. Additionally, voxels where the QUO2 model could not be solved were excluded when performing the ROI analysis of *M*, OEF and CMRO_2_. The resultant ROI probability masks were used to perform weighted averaging of the different measurements and estimates.

#### Registration

Individual ΔR2*_HO_, *M*, OEF and CMRO_2_ maps were non-linearly registered to the ICBM152 template using the CIVET software package [26] via the CBRAIN tool [27] with 12 degrees of freedom, as in Lajoie et al [6]. Test-averaged maps of ΔR2*_HO_ were computed as arithmetic means using in-house software. Averaged maps of *M*, OEF and CMRO_2_ were obtained excluding any voxels where the QUO2 model could not be solved.

### Analysis of sensitivity of model-derived QUO2 values to change in O_2_ concentration

The end-tidal O_2_, blood flow and R2* measurements during a hyperoxia manipulation depend on the employed O_2_ concentration. It was discussed that hyperoxia may also perturb the metabolism [28], however, in our model, we consider HO as an isometabolism challenge as assumed in numerous previous calibrated BOLD studies [1–3]. In order to understand the impact of lower and higher levels of HO (respectively LHO and HHO) to QUO2, we performed an analysis of the sensitivity of its model-derived parameters, *M*, OEF and CMRO_2_, to changes in ETO_2_, CBF and ΔR2*_._ Employing the GM group-average values in Test A during the LHO protocol, we kept constant the parameters not influenced by the O_2_ concentration, while individually varying ETO2_HO_, CBF_HO_ and ΔR2*_HO_ within their respective range delimited by GM group-average values in Test A under each HO protocol, to compute the resultant *M*, OEF and CMRO_2_.

### Statistical analysis

For each model-derived estimate (*M*, OEF and CMRO_2_), we carried out a statistical analysis, using Matlab, on three different combinations of tests: 1) comparing Test A and Test B under the LHO protocol; 2) comparing Test A and Test B under the HHO protocol; 3) comparing tests A between both protocols. When needed, a two-tailed paired t-test was performed, considering a *P* < 0.05 level of significance, to detect any significant difference between tests and protocols. Within each protocol, we also investigated any difference across ROIs by pooling tests values and using family-wise error (FWE) correction for multiple comparisons, set at *P* < 0.05.

Prior to the analysis, statistical tests were performed on the data to ensure it satisfied the repeatability criteria: each distribution of difference between tests was evaluated for normality using the Shapiro-Wilk W-test, while the independence between the magnitude of difference and mean of measurements was verified using a rank correlation coefficient (Kendall’s τ). If the difference distribution appeared to deviate from a normal distribution, or if the magnitude of difference increased with the mean of measurements, the data were transformed on the log_10_ scale and the verification was repeated. In cases where the log_10_ scaled data satisfied the criteria, the repeatability was assessed on these scaled values. Otherwise, assessment of repeatability was based on the original values, as done in previous studies [29–32].

The next metrics were evaluated:

1. dSD, the standard deviation of the difference between tests measurements.
2. wsSD, the within-subject standard deviation, equals dSD/√2 considering two measurements.
3. wsCV, the within-subject (or intra-subject) coefficient of variation, as used in Floyd et al [30] and Chen et al [32]. wsCV = √[mean of the (wsSD/subject mean)^2^]. wsCV provides an unbiased measure of variability expressed as a percent of the mean with a low wsCV indicating a high reproducibility/repeatability. When data were on the log_10_ scale, wsCV was approximated by 10^(wsSD)-1 [33].
4. bsCV, the between-subject (or inter-subject) coefficient of variation as computed in Tjandra et al [34]. bsCV = SD_pooledData_ / mean_pooledData_ * 100.

## Results

One participant reported a high level of anxiety during Test A of the LHO protocol, and the measured CBF response to CO_2_ was found to be twice the standard deviation of the group mean. Data from this participant has been excluded from the present analysis (as in the previous related work [6]).

### Gas manipulation

The test-average and standard deviation of end-tidal O_2_ and CO_2_ at baseline and during periods of hyperoxia are presented in Fig 1. No difference was found within and between protocols resting ETO_2_ (within-protocol: TestA_LHO_ = 112±7 mmHg vs. TestB_LHO_ = 112±3 mmHg, *P* = 0.88, TestA_HHO_ = 113±7 mmHg vs. TestB_HHO_ = 108±7 mmHg, *P* = 0.05; between-protocol: LHO = 112±5 mmHg vs. HHO = 111±7 mmHg, *P* = 0.7). Within-protocol ETO2_HO_ were identical (TestA_LHO_ = 366±6 mmHg vs. TestB_LHO_ = 371±14 mmHg, *P* = 0.37; TestA_HHO_ = 656±17 mmHg vs. TestB_HHO_ = 652±25 mmHg, *P* = 0.42), whereas, as expected, between-protocol ETO2_HO_ were found to be significantly different (LHO = 369±10 mmHg vs. HHO = 654±20 mmHg, *P* < 6*10^−12^). No difference was detected in between-protocol resting ETCO_2_ (LHO = 40±2 mmHg vs. HHO = 42±2 mmHg, *P* = 0.3), nor within the LHO protocol (TestA_LHO_ = 41±2 mmHg vs. TestB_LHO_ = 40±2 mmHg, *P* = 0.57). However a significant difference in resting ETCO_2_ was observed between Test A and Test B under the HHO protocol (TestA_HHO_ = 43±2 mmHg vs. TestB_HHO_ = 40±2 mmHg, *P* < 0.002). This difference in resting ETCO_2_ is in agreement with a lower respiratory rate during Test A compared to Test B (TestA_HHO_ = 6±2 breaths per minute vs. TestB_HHO_ = 8±1 breaths per minute, *P* = 0.03). The ETCO_2_ changes observed during periods of hyperoxia were found to be equivalent within protocol. For the LHO protocol, they were: TestA_LHO_ = -0.8±1.0 mmHg and TestB_LHO_ = -1.1±1.1 mmHg (*P* = 0.8), while for the HHO protocol they were: TestA_HHO_ = -2.5±0.7 mmHg and TestB_HHO_ = -2.4±0.7 mmHg (*P* = 0.5). The averaged decreases in ETCO_2_ were significantly (*P* < 0.005) larger in HHO compared to LHO protocol (LHO = -1.0±1.0 mmHg vs. HHO = -2.4±0.7 mmHg).

### Susceptibility artifacts

**Fig 2:**
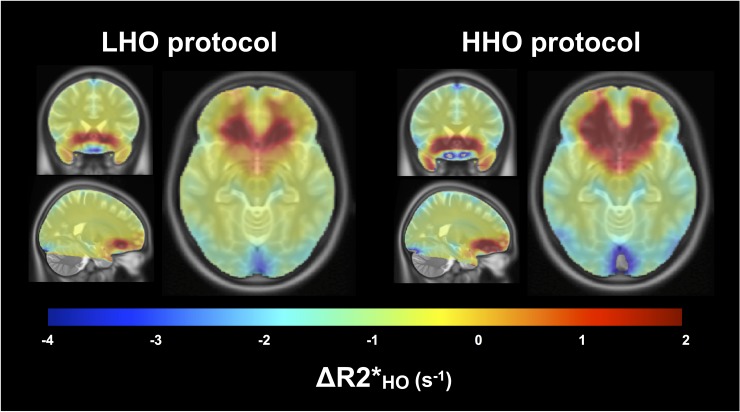
Susceptibility artifacts. For each protocol, the averaged maps of ΔR2* during HO are shown in coronal, sagittal and axial views, overlaying the ICBM152 template. The chosen contrast window facilitates the localization of voxels where an increase in R_2_* is observed (in orange and red). These increases in the transverse relaxation rate are most likely the results of susceptibility artifacts attributable to the presence of paramagnetic O_2_ in frontal sinuses and nasal cavity.

Fig 2 shows a qualitative examination of R2* changes during periods of HO (ΔR2*) through axial, sagittal and coronal views chosen in order to observe regions vulnerable to susceptibility artifacts. No masking, nor median filtering was performed on the functional maps prior to the non-linear registration to the ICBM template and maps average. The contrast window was chosen to facilitate the observation of increase in R2* characterized by orange and red colors. An overall R2* decrease (equivalent to a BOLD increase) in white and gray matter during HO is observed, which is more significant under the more extreme levels of HO. On the other hand, as a repercussion of the presence of paramagnetic oxygen molecules in inhaled air, both protocols presented comparable regions of susceptibility artifacts characterized by positive ΔR2* in voxels surrounding the nasal cavity. Percent of voxels in GM characterized by this increase were found to be the same in both protocols, with 12.8% under the LHO protocol and 11.7% under the HHO protocol (*P* = 0.25), although the positive values were generally higher under the HHO protocol (shown by darker red color). Any voxel affected by the susceptibility artifacts, later results in a non-solution voxel for *M*, OEF and CMRO_2_, and were therefore excluded from the analysis as mentioned in the methodology section.

### *T*_1_ shortening

**Fig 3:**
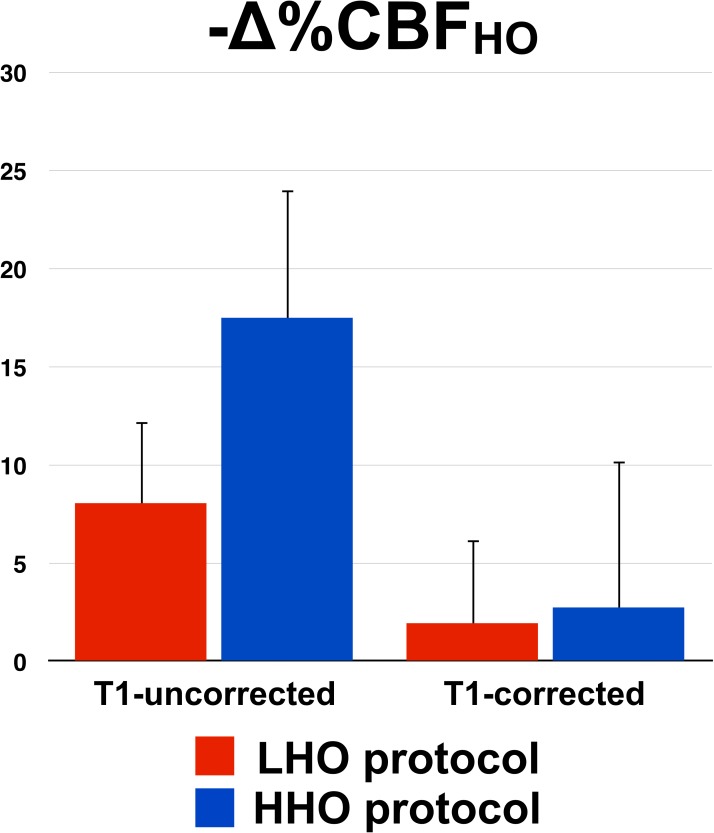
*T*_1_ shortening. For each protocol, pre- and post-*T*_1_-correction CBF changes during HO, averaged across tests, are presented with the standard deviation as error bars.

A value of 1.65 sec was assumed for the normoxic arterial blood *T*_1_ [35], whereas the estimated blood *T*_1_ shortening was larger during the high O_2_ hyperoxia state than during the low hyperoxia challenge: *T*_1HHO_ = 1.47±0.01 sec vs. *T*_1LHO_ = 1.56±0.01 sec, *P* < 4*10^−13^. Fig 3 summarizes, in both protocols, the GM tests average and standard deviation of blood flow decrease during HO before and after correction of blood *T*_1_. While uncorrected, CBF_HO_ decrease was found to be significantly larger under the HHO protocol (LHO = -8.1±4.2 mmHg, HHO = -17.5±6.6 mmHg, *P* < 0.002). After *T*_1_ correction, CBF_HO_ decreases were less pronounced in both protocols, and were not found significantly different from each other (LHO = -1.9±4.3 mmHg, HHO = -2.8±7.5 mmHg, *P* = 0.7) nor from zero (*P*_LHO_ = 0.4, *P*_HHO_ = 0.3).

### Analysis of sensitivity of model-derived QUO2 values to change in O_2_ concentration

**Fig 4:**
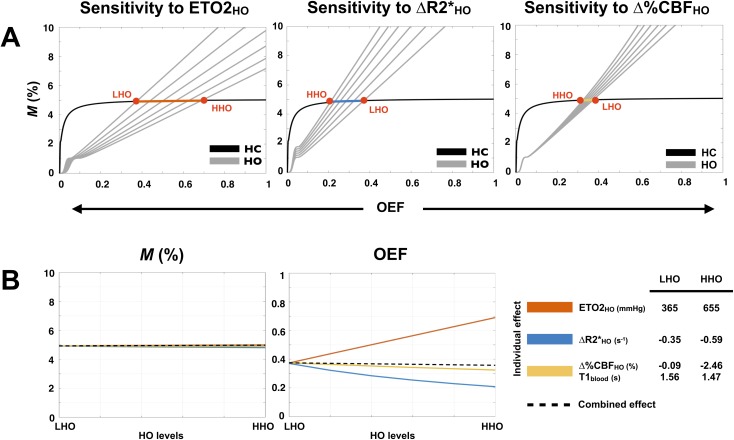
Analysis of sensitivity to O_2_ concentration. Observed effects, on *M* and OEF, of changes in ETO_2_, R2* and CBF following a transition from a low level of hyperoxia (LHO) to a higher level of hyperoxia (HHO) are summarized. Estimates were based on group-averaged Test A measurements during the LHO manipulation, while ETO_2_, R2* and CBF were varied independently ranging from their respective LHO value to their HHO value (values are specified in the legend, with the corresponding blood *T*_1_ below CBF_HO_). The hypercapnia (HC) and hyperoxia (HO) curves resulting from the use of six different values of ETO_2_, R2* and CBF are presented (A). Each red dot represents the HC and HO curves intersection (hence one *M* and OEF solution) when either one of the extremity of the observed range is in use. The remaining *M* and OEF solutions lie on the colored line connecting both red dots. *M* and OEF estimates are presented as a function of the individual (colored lines) and combined (dashed black lines) effect of changes in ETO_2_, R2* and CBF (B).

The individual impacts of changes in ETO2_HO_, ΔR2*_HO_ and Δ%CBF_HO_, on *M* and OEF, as a function of the HO levels are examined by numerical simulations. These changes in ETO2_HO_, ΔR2*_HO_ and Δ%CBF_HO_ are dependent on one another and are examined in order to explain the combined impact on *M* and OEF. Results are summarized in Fig 4. Fig 4A shows the displacement in the HO curves caused by the respective variation of ETO2_HO_, ΔR2*_HO_ and Δ%CBF_HO_, while Fig 4B shows the corresponding OEF and *M* solutions as a function of the individual (colored solid lines) and combined (dashed black lines) changes. Since the O_2_ concentration solely modulates the HO curve, which is shifted on the nearly horizontal section of the HC curve, the changes in ETO2_HO_, ΔR2*_HO_ and Δ%CBF_HO_, either individual or combined, have virtually no impact on the *M* estimates. With respect to OEF, the individual impacts appear to cancel each other out, yielding a modest combined effect. The same conclusion stands for CMRO_2_, since it is the result of multiplying OEF by two measurements that are independent of the hyperoxic stimulus, i.e. the resting CBF and the resting arterial O_2_ content. Therefore, in principle, one would expect *M*, OEF and CMRO_2_ to remain stable, regardless of the O_2_ concentration used to produce hyperoxia. The following sections explore this assumption using real values computed in different ROIs, but also on a voxel-wise basis.

### Protocol-averaged estimates in ROIs

**Fig 5:**
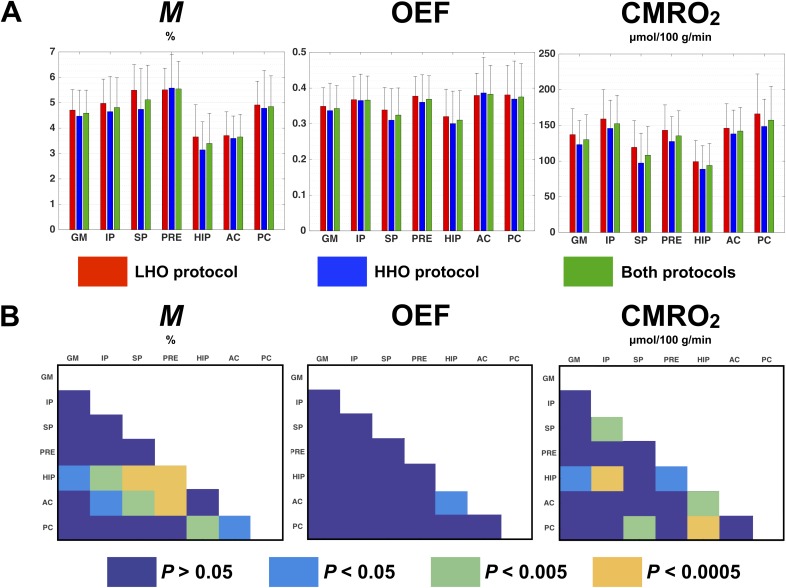
Protocol-averaged estimates in ROIs. *M*, OEF and CMRO_2_ estimates averaged in different ROIs are compared. Fig 5-A presents the ROI-averaged value and standard deviation obtained under the LHO protocol (red bar), the HHO protocol (blue bar) and in both protocols averaged (green bar). Fig 5-B shows, for each estimate, any significant difference observed between ROIs after correcting for multiple comparisons (FWE, *P* < 0.05): dark blue indicates an absence of significant difference (*P* > 0.05), while light blue (*P* < 0.05), green (*P* < 0.005) and orange (*P* < 0.0005) illustrate a significant difference between two ROIs (represented in the X and Y axis). *GM* = gray matter, *IP* = inferior parietal, *SP* = superior parietal, *PRE* = precuneus, *HIP* = hippocampus, *AC* = anterior cingulate, *PC* = posterior cingulate.

In Fig 5A are shown the ROI-averaged *M*, OEF and CMRO_2_ in each protocol (red and blue bars) and over both protocols (green bars). For each combination of model-derived estimate and ROI, we observe a good consistency between protocols with the lowest *P* values being: *P* = 0.17 in superior parietal for *M*, *P* = 0.37 in superior parietal for OEF and *P* = 0.06 in GM for CMRO_2_. Additionally, no apparent divergence was found in variance within each protocol. In Fig 5B are shown, for each estimate, the degree of difference between ROIs, when comparing the estimates averaged over both protocols and correcting for multiple comparisons (FWE set at *P* < 0.05). OEF estimates were found to be similar across ROIs, with the exception between hippocampus and anterior cingulate where a significant difference was detected (*P* = 0.04). Values of *M* and CMRO_2_ in hippocampus were found to be the smallest compared with the other ROIs, with the exception of anterior cingulate (for *M*) and superior parietal (for CMRO_2_).

### Within-subject variability in ROIs

**Fig 6:**
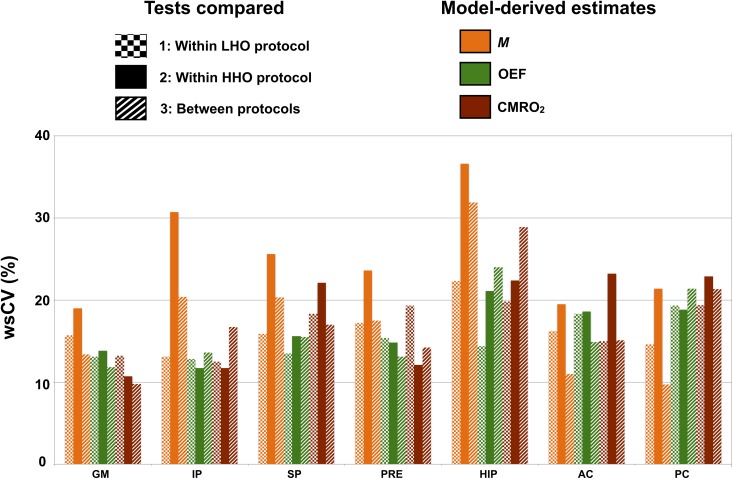
Within-subject variability in ROIs. Computed within-subject CVs (wsCV) are shown for *M*, OEF and CMRO_2_ within each ROI. The model-derived estimates are represented by different colors, while the three combinations of tests are identified by distinct patterns: 1: Test A vs. B under the LHO protocol (squared pattern), 2: Test A vs. B under the HHO protocol (plain pattern), 3: Tests A between both HO protocols (striped pattern). *GM* = gray matter, *IP* = inferior parietal, *SP* = superior parietal, *PRE* = precuneus, *HIP* = hippocampus, *AC* = anterior cingulate, *PC* = posterior cingulate.

Fig 6 presents the within-subject coefficients of variation (wsCV) in every ROIs for *M*, OEF and CMRO_2_. WsCVs were computed for three combinations of tests: 1) test A vs. B under the LHO protocol, 2) test A vs. B under the HHO protocol, 3) tests A between both HO protocols. Across all ROIs, *M* was found to have a lower within-subject variability under the LHO protocol (mean wsCV_LHO_ = 16%, mean wsCV_HHO_ = 25%, *P* = 0.006). On the other hand, within-subject variability of OEF and CMRO_2_ were found unchanged regardless of the HO protocol (OEF: mean wsCV_LHO_ = 15%, mean wsCV_HHO_ = 16%, *P* = 0.2; CMRO_2_: mean wsCV_LHO_ = 17%, mean wsCV_HHO_ = 18%, *P* = 0.6).

### Parametric maps

**Fig 7:**
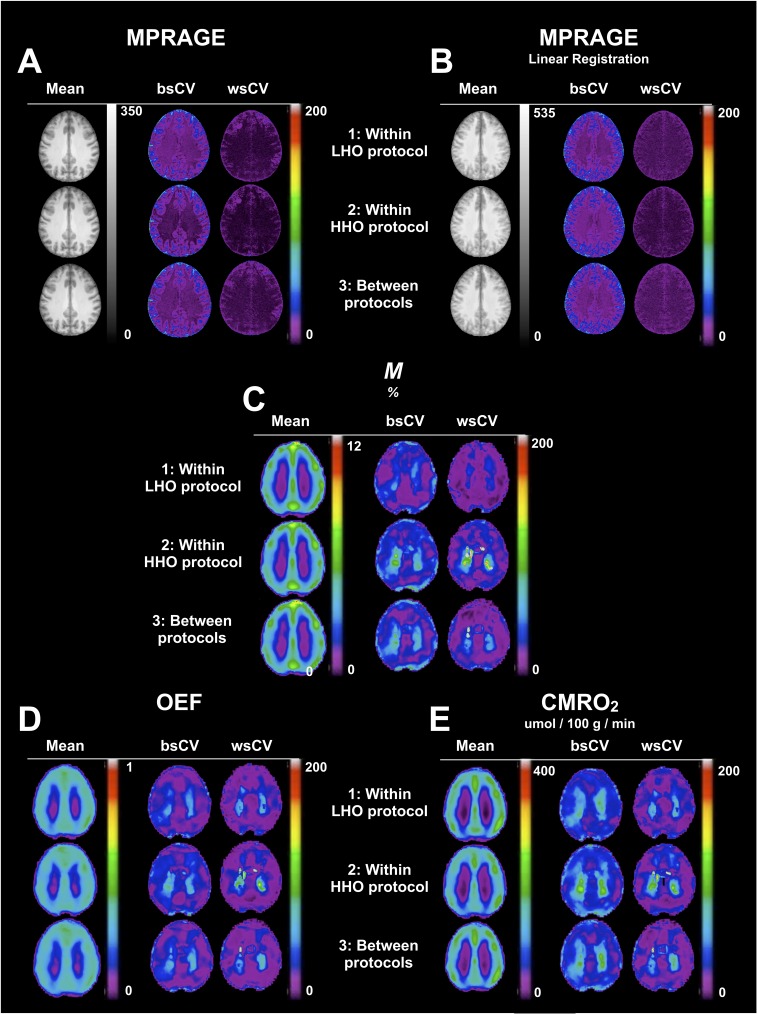
Parametric maps. For each combination of tests (1: Test A vs. B under the LHO protocol, 2: Test A vs. B under the HHO protocol, 3: Tests A between both HO protocols), mean tests, between-subject CV and within-subject CV maps for *M* (C), OEF (D) and CMRO_2_ (E) are shown in one axial slice. Maps were non-linearly registered to the ICBM152 template. As a reference, the equivalent information is presented for MPRAGE maps non-linearly (A) and linearly registered (B) to the template.

In Fig 7, we present, for each combination of tests (1: Test A vs. B under the LHO protocol, 2: Test A vs. B under the HHO protocol, 3: Tests A between both HO protocols), mean tests, between- and within-subject CV maps of *M*, OEF and CMRO_2_. All functional maps were non-linearly registered (NLreg) to the ICBM space. In addition to intrinsic physiological changes, errors in measurements and head movements occurring between the anatomical and the functional scans, a voxel-wise within-subject repeatability may be partly affected by random inaccuracies in registration. In order to evaluate any limitation on the voxel-wise repeatability caused by the registration to the ICBM space, we present the CVs maps for MPRAGE, and verify if any enhancement was possible thanks to the non-linearly registration of our maps (Fig 7A), compared to the linearly registered MPRAGE (Fig 7B). All CVs maps are shown using a window level of 0–200%. At these levels, the passage from 20% to 30% is characterized by the transition from purple to blue, with 30% being an approximate upper limit for what is considered as low variability. Compared to the linearly registered maps (Lreg), the non-linearly registered (NLreg) MPRAGE maps presented a better defined gray matter region, while whole-brain between- and within-subject variability were found to be lower. WsCV values in NLreg were generally found to be <5% in WM, <10% in GM and exceptionally <20% in few small regions, whereas in Lreg wsCV, values were <10% in WM and GM and <20% in with few small regions. Mean maps of *M*, OEF and CMRO_2_ (Fig 7C, 7D and 7E) qualitatively exhibited an absence of dependency on the O_2_ protocol employed. CVs maps of *M* presented slightly less variability under the LHO than the HHO. All three estimates were found to have low GM within-subject variability for the three combinations of tests (<30%). *M* and CMRO_2_ presented a clearer distinction between the population variance and the within-subject variability, whereas OEF was found to have a lower voxel-wise between-subject variability, approaching the within-subject variability.

## Discussion

Performing an analysis of individual impacts, on *M* and OEF, of variation in ETO2_HO_, ΔR2*_HO_ and Δ%CBF_HO_, we have shown how little *M* is affected by the O_2_ concentration in GM, and how the individual impacts on OEF were practically cancelling out, yielding a nearly nonexistent combined impact on OEF and therefore on CMRO_2_. Exploring the within-subject reproducibility in different ROIs as well as on a voxel-wise basis, we observed an unchanged reproducibility for OEF and CMRO_2_ regardless of differences in ETO2_HO_, ΔR2*_HO_ and Δ%CBF_HO_ caused by a distinct O_2_ concentration in inhaled gas. On the other hand, the *M* within-subject repeatability was found to be slightly enhanced under the LHO protocol. No significant difference was found between protocol-averaged values.

In certain situations, the differences between subjects’ brain anatomy are such that a linear transformation is insufficient to register their brain maps on to standard spaces. The local deformations produced by the non-linear registration improve the match. The comparison of linearly versus non-linearly registered individual MPRAGE images provides a qualitative example of the improvement brought by the non-linear registration. The method produced sharper group-averaged maps, characterized by more distinct sulci and more accentuated grey/white matter contrast. Quantitatively, the non-linear co-registration afforded lower CV values.

The presence of paramagnetic molecular oxygen in inhaled air produces susceptibility artifacts. We examined regions vulnerable to those artifacts such as the frontal sinuses and nasal cavity of our ΔR2*_HO_ maps. However, no evidence of enlarged patterns of susceptibility artifacts under inhalation of 100% O_2_ (HHO) compared to 60% (LHO) was found, thus yielding a comparable percent of non-solution voxels in GM for both protocols.

Rather than assuming a fixed value of CBF change during HO, the individual *T*_1_-corrected Δ%CBF_HO_ averaged in GM was used, therefore capturing any intra-subject variation between Test A and Test B in blood flow during HO. Our *T*_1_ values were extrapolated from experimentally-determined values in animal model, which is a common practice in calibrated fMRI approaches. Human blood constitution is similar to that of bovine and rat blood and is likely to experience comparable *T*_1_ shortening during the hyperoxia stimulus [13,36,37]. This is of course an assumption and represents a potential source of confounds in our blood flow changes calculations.

In CBF quantification, so long as the PLD is equal to or higher than the arterial transit time (ATT), the exact ATT value does not matter. In our 2D acquisition, the first and last slices are acquired after a delay of 900 msec and 1986 msec respectively, resulting in a brain-averaged PLD of 1443 msec. Donahue et al. [38] applied a pCASL in a cohort of healthy volunteers (mean age of 30 ± 4 years) and obtained a group-averaged ATT lower than 900 msec within each lobe, including within the occipital lobe with 834 ± 29 msec. We therefore believe that in the large majority of cases, the acquired ASL signal was accurately reflecting CBF and that an increase in our PLD would have resulted in a loss in SNR, especially during hypercapnic where the ATT is known to diminish [38]. Additionally, the ATT increase during HO should be minor as our data indicates that the CBF decreases induced by hyperoxia, even at high O_2_ concentrations, are not substantial. When using a 2D acquisition in a population of elderly or unhealthy patients, it would be recommended to increase the PLD slightly while also imaging a lower number of thicker slices, as in the study De Vis et al. (2015) where a nominal PLD of 1550 msec and 11 slices with 7 mm slice thickness were employed.

Small cohort sizes like that of the present study have been common in recent years, particularly for complex fMRI protocols with greater physiological specificity than the classic BOLD contrast. Despite the relatively small sample size, which limits confidence in the statistical significance of our findings, the present study provides new information on the impact of inspired oxygen levels on calibrated fMRI technique.

To conclude, it was revealed that the pattern of susceptibility artifacts under hyperoxia was comparable regardless of the HO levels. We also demonstrated that variations in ETO2_HO,_ CBF_HO_ and R2*_HO_ were accounted for within the QUO2 model, resulting in an unchanged ROI-averaged *M*, OEF and CMRO_2_ estimates. We observed that the within-subject repeatability was either unchanged (for OEF and CMRO_2_) or slightly enhanced under the LHO protocol (for *M*). In summary, the use of a higher hyperoxic challenge revealed no beneficial impact on the calibrated fMRI measurements, while a reduced concentration of 60% O_2_ was shown to maintain sufficient BOLD contrast and to produce consistent model-derived results.
